# Hsa_circ_0007990 promotes breast cancer growth via inhibiting YBX1 protein degradation to activate E2F1 transcription

**DOI:** 10.1038/s41419-024-06527-7

**Published:** 2024-02-20

**Authors:** Tao Xu, Mengqiu Xiong, Qiwei Hong, Bei Pan, Mu Xu, Ying Wang, Yalan Sun, Huiling Sun, Yuqin Pan, Shukui Wang, Bangshun He

**Affiliations:** 1 General Clinical Research Center, Nanjing First Hospital, Nanjing Medical University, Nanjing, Jiangsu China; 2 Department of Laboratory Medicine, Nanjing First Hospital, Nanjing Medical University, Nanjing, Jiangsu China; 3 Department of Clinical Pharmacy, School of Basic Medicine and Clinical Pharmacy, China Pharmaceutical University, Nanjing, Jiangsu China; 4 Jiangsu Cancer Personalized Medicine Collaborative Innovation Center, Nanjing, Jiangsu China

**Keywords:** Breast cancer, Cell growth

## Abstract

Breast cancer (BC) is the most commonly diagnosed malignant tumour in females worldwide. Although remarkable advances in early detection and treatment strategies have led to decreased mortality, recurrence and metastasis remain the major causes of cancer death in BC patients. Increasing evidence has demonstrated that circular RNAs (circRNAs) play critical roles in cancer progression. However, the detailed biological functions and molecular mechanisms of circRNAs in BC are unclear. The aim of this study was to investigate the possible role of circRNAs in the progression of BC. Differentially expressed circRNAs in BC were identified by integrating breast tumour-associated somatic CNV data and circRNA high-throughput sequencing. Aberrant hsa_circ_0007990 expression and host gene copy number were detected in BC cell lines via quantitative polymerase chain reaction (qPCR). The expression level of hsa_circ_0007990 in BC tissues was validated by in situ hybridization (ISH). Loss- and gain-of-function experiments were performed in vitro and in vivo, respectively, to explore the potential biological function of hsa_circ_0007990 in BC. The underlying mechanisms of hsa_circ_0007990 were investigated through MS2 RNA pull-down, RNA immunoprecipitation, RNA fluorescence in situ hybridization, immunofluorescence, chromatin immunoprecipitation and luciferase reporter assays. The levels of hsa_circ_0007990 were elevated in BC tissues and cell lines, an effect that was partly due to host gene copy number gains. Functional assays showed that hsa_circ_0007990 promoted BC cell growth. Mechanistically, hsa_circ_0007990 could bind to YBX1 and inhibit its degradation by preventing ubiquitin/proteasome-dependent degradation, thus enhancing the expression of the cell cycle-associated gene E2F1. Rescue experiments suggested that hsa_circ_0007990 promoted BC progression through YBX1. In general, our study demonstrated that hsa_circ_0007990 modulates the ubiquitination and degradation of YBX1 protein and further regulates E2F1 expression to promote BC progression. We explored the possible function and molecular mechanism of hsa_circ_0007990 in BC and identified a novel candidate target for the treatment of BC.

## Introduction

Breast cancer (BC) has become the most commonly diagnosed cancer worldwide [1]. In recent years, the mortality of BC has decreased significantly due to early detection and improvements in treatment. However, recurrence and metastasis still occur frequently and continue to threaten survival [2]. Since the molecular mechanisms of BC development are poorly understood, additional investigations are needed to discover novel pathogenic factors and therapeutic targets.

Circular RNAs (circRNAs) are special kinds of RNA with covalently closed and single-stranded circular structures generated from pre-mRNA back-splicing by various mechanisms [3]. Numerous studies have shown that circRNAs are highly conserved, stable, and abundant and exhibit disease- and tissue-specific expression patterns, which enable them to perform vital biological functions in multiple diseases, including cancers [4]. Accumulating evidence shows that dysregulated circRNAs, such as the gastric cancer suppressor circMTHFD2L [5], the colorectal cancer progression promotor circINSIG1 [6] and BC-overexpressing circPVT1 [7], can act as oncogenic and tumour suppressors. Additionally, circRNAs reportedly participate in the pathogenesis of cancers through various regulatory mechanisms involving miRNA decoys, protein scaffolds, translation templates and alternative splicing regulators [4]. Although some progress has been made in the study of circRNAs, the roles of circRNAs in BC tumorigenesis and the detailed molecular mechanisms involved need to be further elucidated.

Herein, we found that a novel circRNA hsa_circ_0007990, derived from exon 2 and exon 3 of the PGAP3 gene, is upregulated in BC tissues and cell lines. The knockdown of hsa_circ_0007990 suppressed the capacity of BC cells growth. Mechanistically, hsa_circ_0007990 interacted with YBX1 and inhibited its ubiquitination and degradation, subsequently promoted E2F1 transcription. Our research contributes to understanding the mechanisms of BC progression and provides a potential therapeutic target for BC in the future.

## Materials and methods

### BC tissue samples, cell lines, and cell culture

A tissue microarray (TMA) containing forty pairs of BC tissues and adjacent non-tumor tissues was collected from Nanjing First Hospital, and all patients provided signed informed consent for the use of their tissues for scientific research. Clinicopathological parameters of the patients, including gender, age, location, size, stage, and lymph node metastasis, were obtained. A normal human mammary epithelial cell line (MCF-10A) and BC cell lines (MCF-7, MDA-MB-231, T47D, BT549 and MDA-MB-468) were obtained from the Shanghai Institute of Cell Biology. These cell lines were cultured with recommended media and conditions according to the instructions provided. The culture method used for MCF-10A cells was described previously [8]. MCF-7 and BT549 cells were grown in Roswell Park Memorial Institute (RPMI; KeyGEN, China) 1640 medium supplemented with 10% fetal bovine serum (FBS; ABW, Uruguay). MDA-MB-231 and MDA-MB-468 cells were maintained in Leibovitz’s L-15 (L-15; KeyGEN, China) medium supplemented with 10% FBS. RPMI 1640 supplemented with 10% FBS and 0.02 mg/ml insulin (Beyotime, China) was used to culture the T47D cells. All cell lines were grown at 37 °C in a 5% CO_2_ cell incubator.

### RNA chromogenic in situ hybridization (CISH)

The expression of hsa_circ_0007990 was detected by TMA using a specific digoxigenin-labelled probe. The detailed sequence of this probe is listed in Supplementary Table S1. Following the manufacturer’s protocol, the tissues were digested with proteinase K after dewaxing and rehydration, fixed in 4% paraformaldehyde and incubated with the specific digoxigenin-labelled probe overnight at 55 °C. Then, the tissues were incubated overnight at 4 °C with an anti-DIG-HRP antibody. The sections were stained with diaminobenzidine and hematoxylin. Finally, the CISH-stained tissue sections were analysed with the Aipathwell software (Servicebio, Wuhan, China). The intensity of the positive staining (0, negative; 1 + , weak; 2 + , moderate; and 3 + , strong) was recorded. The final H-scores were calculated using the following formula: [1 × (% cells 1 + ) + 2 × (% cells 2 + ) + 3 × (% cells 3 + )] × 100.

### RNA and gDNA isolation and quantitative polymerase chain reaction (qPCR)

Total RNA was isolated from tissues and cells with TRIzol reagent (Invitrogen, USA) following the manufacturer’s protocol. Genomic DNA (gDNA) was extracted by using a TIANamp Genomic DNA Kit (TIANGEN, China). RNA isolation from nuclear and cytoplasmic fractions was performed with the PARIS Kit (Ambion, Life Technologies) following the manufacturer’s protocol. The expression of circRNAs and mRNAs was assessed with an Applied Biosystems 7500 Sequence Detection System. GAPDH expression was normalized to the internal gene. The sequences of all primers used in this study are listed in Supplementary Table S1.

### Plasmid construction and cell transfection

The lentiviruses used for hsa_circ_0007990 knockdown and overexpression and the corresponding control were purchased from Genechem (Shanghai, China). BC cells were infected with lentivirus following the manufacturer’s instructions. The full-length complementary cDNA of human YBX1 was synthesized and subcloned and inserted into the pcDNA3.1 expression vector by Kingsray Biotechnology (Nanjing, China). The plasmid was transfected into BC cells using ExFect^®^ Transfection Reagent (Vazyme, Nanjing). All the sequences are listed in Supplementary Table S1.

### Agarose gel electrophoresis

The PCR products of cDNA and gDNA were separated by using 4% agarose gel electrophoresis with TBE buffer solution and GelRed (GLPBIO, USA) at 110 V for 30 min. A DL500 DNA marker (Takara, Japan) was used, and these bands were detected by UV irradiation (EC3-300 Imaging System, USA).

### Fluorescence in situ hybridization assay (FISH)

FISH assay was performed with a Fluorescent In Situ Hybridization Kit (Ribobio, Guangzhou) following the manufacturer’s protocols. The hybridization was carried out using Cy3-labelled hsa_circ_0007990 probes (Ribobio, Guangzhou). Fluorescence images were captured under a Zeiss microscope (Carl Zeiss Microscopy GmbH, China).

### RNase R treatment

Total RNA from BC cells was treated with RNase R following the manufacturer’s instructions (Geneseed, Guangzhou). Then, the expression of hsa_circ_0007990 and PGAP3 mRNA was detected by qPCR and normalized to that of GAPDH.

### Cell proliferation and cell cycle assays

Cell proliferation assay was assessed with Cell Counting Kit-8 (CCK8; Vazyme, Nanjing), colony formation assay and EdU assay kits (Ribobio, Guangzhou) following the manufacturer’s instructions. For the cell cycle assay, BC cells were stained with a Cell Cycle Detection Kit (KeyGEN, Jiangsu) following the manufacturer’s manual and analysed with a DxFLEX flow cytometer (Beckman Coulter, USA). The results were analysed by ModFit LT software.

### Animal experiments

For tumour growth assays in vivo, female BALB/c nude mice (six weeks old) were randomly divided and raised under pathogen-free conditions following the protocols approved by the ethics committee of Nanjing First Hospital. Hsa_circ_0007990 knockdown, overexpression, and negative control transfected MCF-7 and MDA-MB-231 cells were harvested and subcutaneously injected into two flanks of each nude mouse in the armpit. The tumour size was measured weekly, and all the mice were euthanized after five weeks or four weeks. The tumours were collected, weighed, and further analysed by HE and IHC staining. The sample size is described in the corresponding figure legend. The investigator was blinded to the group allocation of the mice during the experiment.

### MS2 RNA pull-down assay

The hsa_circ_0007990, hsa_circ_0007990-MS2 and MS2-CP overexpression vectors were constructed by Geneseed (Guangzhou, China). MDA-MB-231 cells were co-transfected with hsa_circ_0007990-MS2 or hsa_circ_0007990 and the MS2-CP overexpression vector using Lipofectamine 2000 (Invitrogen, USA). After 48 h, the cell lysates were incubated with anti-CP antibody and proteinA+G magnetic beads at 4 °C overnight. The proteins pulled down were collected with elution buffer and further analysed by mass spectrometry and western blotting.

### RNA immunoprecipitation (RIP)

A Magna RIP^TM^ RNA-binding protein immunoprecipitation kit (Millipore, USA) was used for RNA immunoprecipitation (RIP) following the manufacturer’s instructions. In brief, BC cells were lysed with RIP lysis buffer and incubated with magnetic beads coated with anti-YBX1 (ab76149, Abcam) or control anti-IgG (ab172730, Abcam) antibodies overnight at 4 °C. Then, the beads were washed and treated with Proteinase K. Finally, the bound RNA was isolated and analysed via qPCR.

### Immunofluorescence (IF) assay

Cells seeded on the slides in 24-well plates were fixed with 4% paraformaldehyde at room temperature for 15 min and washed three times with PBS. The cells were permeabilized with 0.5% Triton X-100 for 20 min and then blocked with 5% BSA solution for 30 min. The cells were incubated with diluted anti-YBX1 (ab76149, Abcam) antibody overnight at 4 °C. The next day, the slides were incubated with a secondary antibody and stained with DAPI (Santa Cruz Biotechnology, USA). Images were acquired with a Zeiss microscope (Carl Zeiss Microscopy GmbH, China).

### Cycloheximide (CHX) and MG132 treatment

Hsa_circ_0007990 knockdown or negative control cells were cultured with cycloheximide (CHX, 200 μg/ml) (Selleck, USA), which inhibits mRNA translation. The cells were harvested and lysed with lysis buffer at 0, 4, 8 and 12 h. For MG132 treatment, cells were incubated with the proteasome inhibitor with MG132 (20 μM, MCE, China) for 4 h. The degradation of YBX1 protein was subsequently measured via western blotting.

### Chromatin immunoprecipitation (ChIP) assay

A SimpleChIP^®^ Enzymatic Chromatin IP Kit (Magnetic Beads) (Cell Signaling Technology, USA) was used following the manufacturer’s protocol. Briefly, MCF-7 cells were cross-linked and sonicated to obtain DNA fragments ranging from 200 to 400 bp in length. Then, the lysates were incubated with anti-YBX1 (ab76149, Abcam) or anti-IgG (ab172730, Abcam) antibodies overnight at 4 °C. Finally, immunoprecipitated DNA was isolated and analysed via qPCR. The specific ChIP-qPCR primers used are listed in Supplementary Table S1.

### Luciferase reporter assay

For the E2F1 promoter luciferase reporter assay, wild-type or mutant plasmid with YBX1 binding sites validated by ChIP were constructed and subcloned and inserted into pGL3-basic vectors (GeneCreat, China). The pRL-TK vector was used as a control, and luciferase activity was assessed using a Dual-Lumi^TM^ Luciferase Reporter Gene Assay Kit (Beyotime, China).

### Protein extraction and western blotting (WB)

Cell protein lysates were harvested using a protein extraction kit (KeyGEN, China). The concentration of protein was measured with a BCA protein quantitation assay (KeyGEN, China). The detailed procedures were performed following protocols described previously [9]. Anti-GAPDH (Cat No. 10494-1-AP) and anti-Ki67 (Cat No. 27309-1-AP) antibodies were purchased from Proteintech (China). Anti-YBX1 (ab76149) and anti-ubiquitin (ab134953) antibodies were purchased from Abcam (USA). Anti-E2F1 (#3742) antibody was purchased from Cell Signaling Technology (USA). Goat Anti-Rabbit IgG, Peroxidase Conjugated, H + L (BL003A) was purchased from BioSharp (China).

### Statistical analysis

All the data were analysed with SPSS 22.0 software (IBM, USA), and figures were generated with GraphPad Prism 7.0 software (GraphPad Software, USA). Results with normal distribution were given as mean ± standard deviation (SD). Differences between groups were determined by Student’s *t* test or one-way ANOVA. All tests were two-sided and *P* < 0.05 was considered to indicate statistical significance. All the experiments were carried out at least three times.

## Results

### Identification of a novel hsa_circ_0007990 significantly upregulated in breast cancer

Copy number variation (CNV) is considered an important driver of tumorigenesis [10]. Many cancer-driving CNV loci that encode proteins and non-coding transcripts have been identified [11–13]. Herein, we integrated previously published human breast tumour-associated somatic CNV data [14] and circRNA high-throughput sequencing data generated by our team [15]. Among the 20 most significantly changed circRNAs in our previous study, circPGAP3 (circBase ID: hsa_circ_0007990), which was upregulated in BC samples and whose host gene PGAP3 was at the 17q12 amplification locus, attracted our attention (Supplementary Fig. S1).

**Fig. 1:**
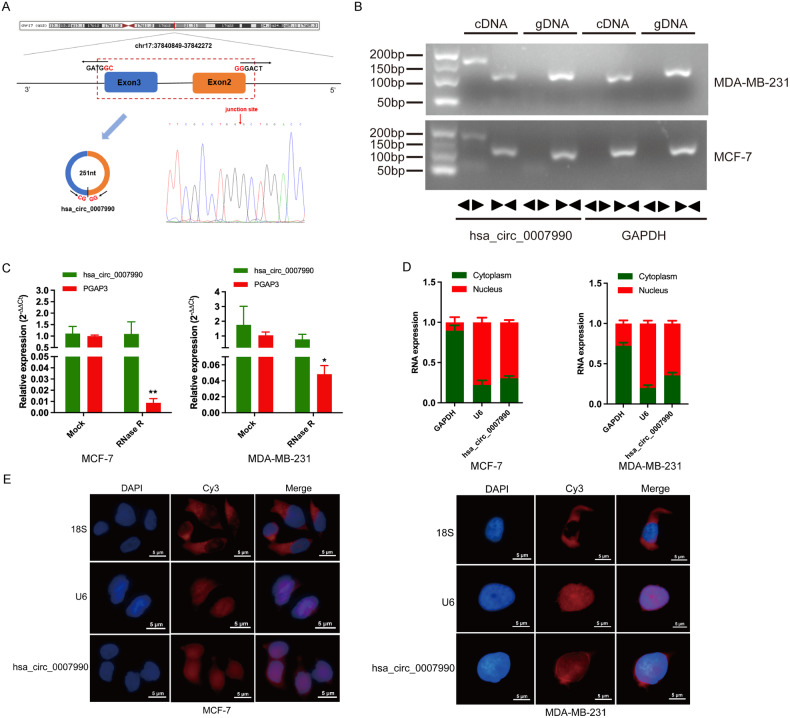
Characterization of hsa_circ_0007990. **A** Schematic illustration of hsa_circ_0007990 originating from the PGAP3 gene on chromosome 17 and the head-to-tail splicing site was confirmed by Sanger sequencing. **B** The existence of hsa_circ_0007990 was detected in cDNA but not in gDNA using divergent primers by RT-PCR and electrophoresis analysis. GAPDH was used as a control. **C** The expression of hsa_circ_0007990 and its host gene PGAP3 mRNA after RNase R treatment were detected by qPCR. **D**, **E** The nuclear-cytoplasmic separation experiment and fluorescence in situ hybridization (FISH) assay were performed to detect the subcellular localization of hsa_circ_0007990. U6 and GAPDH or 18 S were used as positive controls in the nucleus and cytoplasm, respectively. Hsa_circ_0007990 probe was labeled with Cy3 (red), nuclei were stained with DAPI (blue). Scale bar = 5 μm. *P < 0.05, **P < 0.01.

According to the UCSC Genome Brower (http://genome.ucsc.edu), we identified that hsa_circ_0007990 was generated by back-splicing between exon 2 and exon 3 of the PGAP3 gene (17q12, chr17:37840849-37842272), which is 251 nt in length. The head-to-tail splicing site was confirmed by Sanger sequencing of the PCR products (Fig. 1A). Subsequently, the characteristics of hsa_circ_0007990 were further demonstrated in BC cells. We designed divergent primers and convergent primers to detect circular and linear transcripts, respectively. Agarose gel electrophoresis revealed that hsa_circ_0007990 was detectable only when divergent primers were used for cDNA and not when genomic DNA (gDNA) was used (Fig. 1B). Linear GAPDH was used as a control. To evaluate the stability of hsa_circ_0007990, we treated BC cells with RNase R (a 3’ to 5’ exoribonuclease that degrades linear RNA) and found that, compared with linear PGAP3 mRNA, hsa_circ_0007990 was resistant to RNase R (Fig. 1C). These results confirmed the loop structure of hsa_circ_0007990. Additionally, a nuclear-cytoplasmic separation experiment and fluorescence in situ hybridization (FISH) assay were performed to determine the subcellular localization of hsa_circ_0007990. The results consistently showed that hsa_circ_0007990 was predominantly located in the nucleus (Fig. 1D, E).

**Fig. 2:**
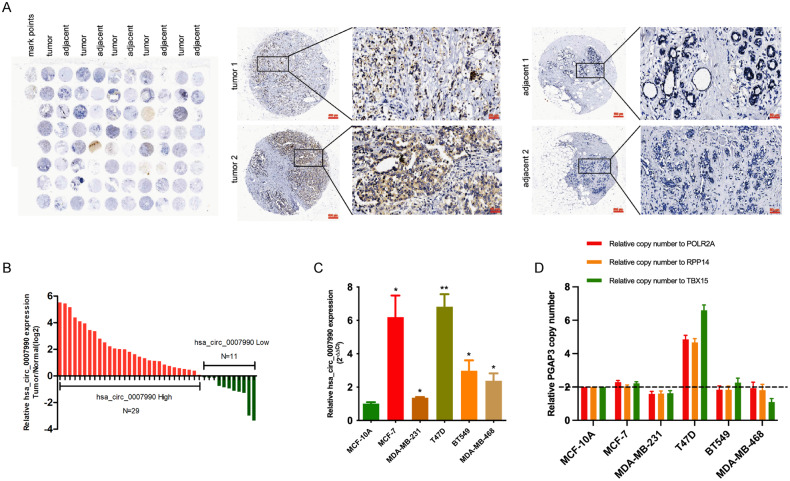
Hsa_circ_0007990 was significantly upregulated in breast cancer. **A** ISH analysis detecting hsa_circ_0007990 expression in BC tissue (*n* = 40) and adjacent normal tissues (*n* = 40) (left). Representative images of two BC tissues and adjacent normal tissues were shown (right). **B** The expression ratio of hsa_circ_0007990 in 40 paired tissues of BC. Red: Log2 (T/N expression) value > 1; Green: Log2 (T/N expression) value ≤ 1. **C**, **D** The expression of hsa_circ_0007990 and the copy number of its host gene PGAP3 were measured in the normal mammary epithelial cell line MCF-10A and BC cell lines via qPCR analysis. Scale bar = 50 μm or 500 μm. *P < 0.05, **P < 0.01.

To validate the expression level of hsa_circ_0007990 in BC, we designed and synthesized in situ hybridization (ISH) probes for hsa_circ_0007990 and measured its expression in 40 pairs of BC tissues and their adjacent normal tissues (Fig. 2A). Analysis of the tumour tissue microarray indicated that hsa_circ_0007990 was upregulated in 29/40 BC tissues, and the expression was 6.61-fold greater on average (*P* = 0.002) (Fig. 2B). In addition, the expression of hsa_circ_0007990 was greater in patients with larger tumours, lymph node metastasis and high Ki67 expression and in the ER- subgroup (Supplementary Fig. S2A–D), although there was no significant difference due to the small sample size. Furthermore, we analysed the level of hsa_circ_0007990 in BC cells and found that it was significantly greater than that in the normal mammary epithelial cell line MCF-10A (Fig. 2C). Additionally, qPCR revealed that the host gene PGAP3 copy number was gained in MCF-7 and T47D cells (Fig. 2D), suggesting that the upregulation of hsa_circ_0007990 is partly due to PGAP3 gene amplification.

### Hsa_circ_0007990 promotes the growth of BC in vitro and vivo

**Fig. 3:**
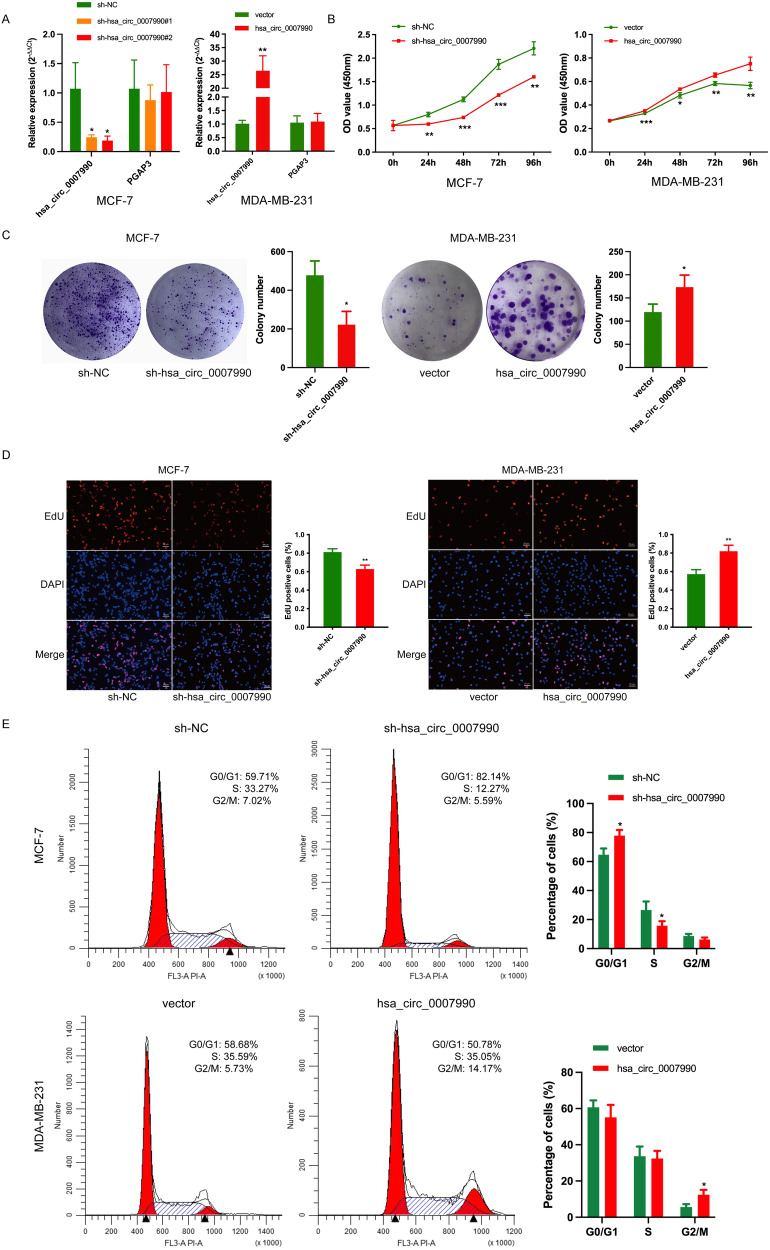
Hsa_circ_0007990 promotes the growth of BC in vitro. **A** The expression of hsa_circ_0007990 and host gene PGAP3 mRNA in BC cells transfected with sh-hsa_circ_0007990, overexpression vector, and corresponding controls were analyzed by qPCR. Hsa_circ_0007990 promotes the proliferation of BC cells performed by **B** CCK8, **C** colony formation, **D** EdU, and **E** cell cycle assays. Scale bar = 20 μm. *P < 0.05, **P < 0.01, ***P < 0.001.

To explore the potential biological function of hsa_circ_0007990 in BC, we designed two knockdown sequences (Supplementary Table S1) targeting the back-splice junction site of hsa_circ_0007990 and transduced them into MCF-7 cells through lentivirus (Supplementary Fig. S3A). The qPCR results showed that the knockdown efficiency of sh-hsa_circ_0007990#2 was the most significant and that the level of the host gene PGAP3 did not change (Fig. 3A). Therefore, we used the sh-hsa_circ_0007990#2 sequence to silence hsa_circ_0007990 in our subsequent functional experiments. Additionally, we constructed an overexpression vector for hsa_circ_0007990 in MDA-MB-231 cells through lentivirus-mediated transduction (Supplementary Fig. S3B), and the overexpression efficiency of hsa_circ_0007990 was confirmed (Fig. 3A). Then, we performed functional experiments to observe the biological behavior of the BC cells. CCK8 assays showed that hsa_circ_0007990 knockdown significantly suppressed the proliferation of MCF-7 cells, while hsa_circ_0007990 overexpression markedly increased MDA-MB-231 cell proliferation (Fig. 3B). The colony formation assays further supported these results (Fig. 3C). In addition, the EdU assays demonstrated that DNA synthesis in MCF-7 cells could be inhibited by silencing hsa_circ_0007990, whereas the upregulation of hsa_circ_0007990 in MDA-MB-231 cells produced the opposite effect (Fig. 3D). Cell cycle assays revealed that MCF-7 cells transfected with sh-hsa_circ_0007990 were arrested in G0/G1 phase and decreased in S phase, while overexpression of hsa_circ_0007990 resulted in G2/M arrest in MDA-MB-231 cells (Fig. 3E). Taken together, these in vitro experiments suggested that hsa_circ_0007990 could enhance BC cell growth.

**Fig. 4:**
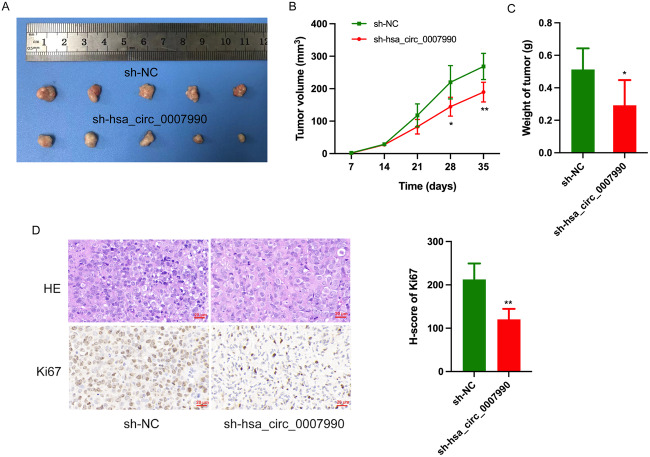
Hsa_circ_0007990 promotes the growth of BC in vivo. **A** Tumor xenograft model in nude mice (MCF-7 cells). **B** A slower growth rate and **C** lower tumor weight was observed in the xenograft tumors treated with sh-hsa_circ_0007990 compared with the control group. **D** Representative images for H&E staining, Ki67 IHC of xenograft tumors in different groups. Sample size *n* = 5 for each group. Scale bar = 20 μm. *P < 0.05, **P < 0.01.

To further investigate the effect of hsa_circ_0007990 on BC cell growth in vivo, xenograft tumours in nude mice were conducted. As shown in Fig. 4A–C, the xenograft tumours in the hsa_circ_0007990 knockdown group exhibited a slower growth rate and lower tumour weight than did those in the control group. Unsurprisingly, tumours in the hsa_circ_0007990 overexpression group exhibited the opposite effect (Supplementary Fig. S4). IHC staining analysis revealed that the expression of the proliferation marker Ki67 was decreased in the hsa_circ_0007990 knockdown group (Fig. 4D). Therefore, these results indicated that hsa_circ_0007990 could enhance BC cell growth in vivo.

### Hsa_circ_0007990 directly binds to YBX1 and stabilizes the protein

**Fig. 5:**
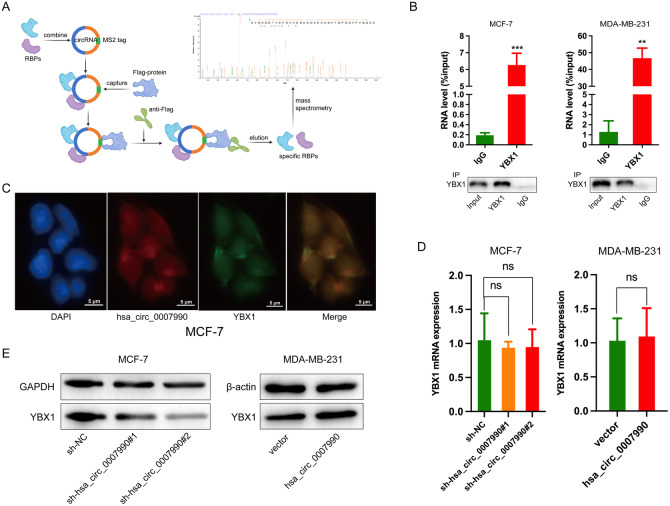
Hsa_circ_0007990 directly binds to YBX1 and stabilizes its protein levels. **A** MS2 RNA pull-down and subsequent mass spectrometry assays were carried out to identify the potential proteins binding to hsa_circ_0007990. **B** RIP assays using an anti-YBX1 antibody was performed in BC cells. IgG was used as negative control. The expression of hsa_circ_0007990 in different groups was detected by qPCR analysis. **C** The co-localization of hsa_circ_0007990 and YBX1 was assessed by FISH-IF. **D**, **E** The mRNA and protein levels of YBX1 were measured by qPCR and western blot after silence and overexpression of hsa_circ_0007990. Scale bar = 5 μm. **P < 0.01, ***P < 0.001.

To elucidate the underlying molecular mechanism of hsa_circ_0007990-induced BC progression, we conducted a series of investigations into the main functions of circRNAs. First, circRNAs exert their biological functions by regulating its host gene expression [3]. After silencing or overexpressing hsa_circ_0007990, we examined the expression of PGAP3 and found that changes in hsa_circ_0007990 expression had no effect on PGAP3 mRNA (Fig. 3A). Second, the best-known function of circRNAs is to act as miRNA sponges to regulate targets via ceRNA mechanism [16]. However, as mentioned above, hsa_circ_0007990 was predominantly located in the nucleus, thus, it seems a little probability that mainly functioned as a ceRNA. Third, studies in recent years have shown that nucleus-retained circRNAs can perform their functions by encoding proteins [17]. Bioinformatic analysis by circBank (http://www.circbank.cn) revealed that hsa_circ_0007990 has no ribosome entry site (IRES), suggesting that the possibility of this circRNA encoding a protein is relatively low (Supplementary Fig. S5). Finally, circRNA-protein interactions may play critical roles in tumour progression [18]. To identify the potential proteins that bind to hsa_circ_0007990, we carried out MS2 RNA pull-down assays and subsequent mass spectrometry assays (Fig. 5A). Among these proteins that specifically interacted with hsa_circ_0007990, Y-box binding protein 1 (YBX1) was selected because of its higher score (Supplementary Table S2). RIP assays using an anti-YBX1 antibody were performed to validate the interaction between hsa_circ_0007990 and YBX1 in BC cells (Fig. 5B). Consistently, FISH-IF analysis revealed that hsa_circ_0007990 co-located with YBX1 in the nucleus (Fig. 5C). Taken together, these findings demonstrated that hsa_circ_0007990 could directly bind to YBX1 in BC cells. Next, whether the expression of YBX1 was regulated by hsa_circ_0007990 was assessed. The qPCR and western blot assays showed that hsa_circ_0007990 did not change the mRNA level of YBX1 but did significantly affect its protein expression. Hsa_circ_0007990 silencing reduced the protein level of YBX1 in MCF-7 cells, whereas hsa_circ_0007990 overexpression enhanced the YBX1 protein level in MDA-MB-231 cells (Fig. 5D, E). These results indicated that hsa_circ_0007990 could stabilize YBX1 protein levels.

### Hsa_circ_0007990 protects YBX1 from ubiquitin/proteasome-dependent protein degradation

**Fig. 6:**
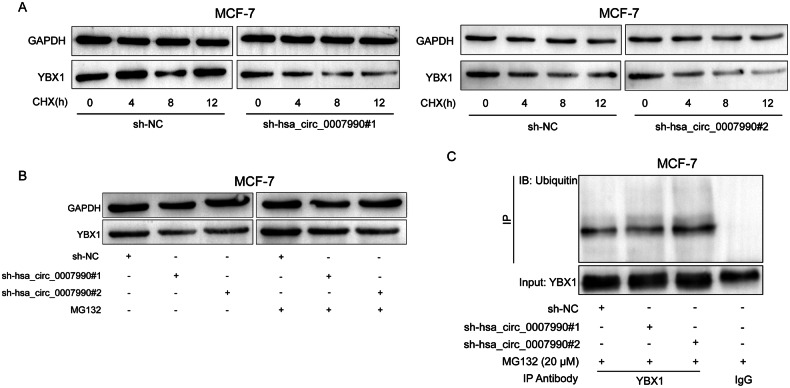
Hsa_circ_0007990 protects YBX1 from ubiquitin/proteasome-dependent protein degradation. **A** Western blot analysis of YBX1 protein expression in MCF-7 cells treated with CHX (200 μg/ml) for the indicated times. **B** Western blot analysis of YBX1 protein expression in MCF-7 cells transfected with or without sh-hsa_circ_0007990 following proteasome inhibition with MG132 treatment (20 μM) for 4 h. **C** Ubiquitination modification of YBX1 was immunoprecipitated from MCF-7 cells transfected with or without sh-hsa_circ_0007990 and treated with MG132 (20 μM) for 4 h.

To explore the role of hsa_circ_0007990 in YBX1 protein regulation, we treated MCF-7 cells with the protein synthesis inhibitor cycloheximide (CHX) and found that YBX1 protein levels decreased more upon hsa_circ_0007990 silencing (Fig. 6A). However, YBX1 protein expression in hsa_circ_0007990-silenced cells treated with MG132 was not obviously reduced compared with that in control cells without MG132 treatment (Fig. 6B). We further investigated whether hsa_circ_0007990 regulates the ubiquitination level of YBX1. Immunoprecipitation (IP) and western blotting showed that hsa_circ_0007990 knockdown significantly increased the ubiquitination level of YBX1 in MCF-7 cells (Fig. 6C). These results suggested that hsa_circ_0007990 might stabilize the YBX1 protein by preventing its ubiquitin/proteasome-dependent protein degradation.

### Hsa_circ_0007990 promotes BC progression through YBX1

**Fig. 7:**
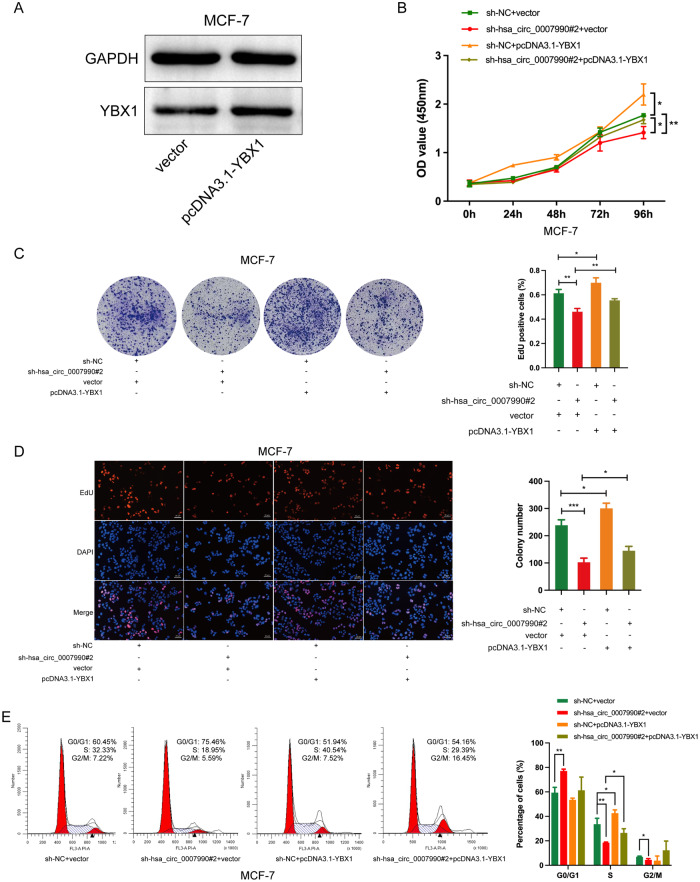
Hsa_circ_0007990 invokes BC progression through YBX1. **A** The protein level of YBX1 was measured by western blot after pcDNA3.1-YBX1 plasmid transfected into MCF-7 cells. Rescue experiments were performed by **B** CCK8, **C** colony formation, **D** EdU, and **E** cell cycle assays. Scale bar = 20 μm. *P < 0.05, **P < 0.01, *** P < 0.001.

YBX1 is a multifunctional DNA- and RNA-binding protein that is a member of the highly conserved family of cold shock proteins. Previous studies have shown that YBX1 is associated with the malignant phenotypes in several types of tumours, including BC [19]. Survival analysis revealed that BC patients with higher YBX1 expression had lower overall survival (OS) and recurrence-free survival (RFS), suggesting an important role for YBX1 in BC (Supplementary Fig. S6A–D). To confirm whether the interaction between YBX1 and hsa_circ_0007990 contributes to BC progression, we successfully transfected the pcDNA3.1-YBX1 plasmid into the MCF-7 cell line (Fig. 7A) and then performed rescue experiments in hsa_circ_0007990-knockdown cells. Unsurprisingly, elevated YBX1 expression partly reversed the inhibition of cell proliferation caused by hsa_circ_0007990 knockdown (Fig. 7B–E). Overall, these results verified that hsa_circ_0007990 invokes BC progression through YBX1.

### Hsa_circ_0007990 regulates E2F1 expression via a YBX1-dependent pathway

**Fig. 8:**
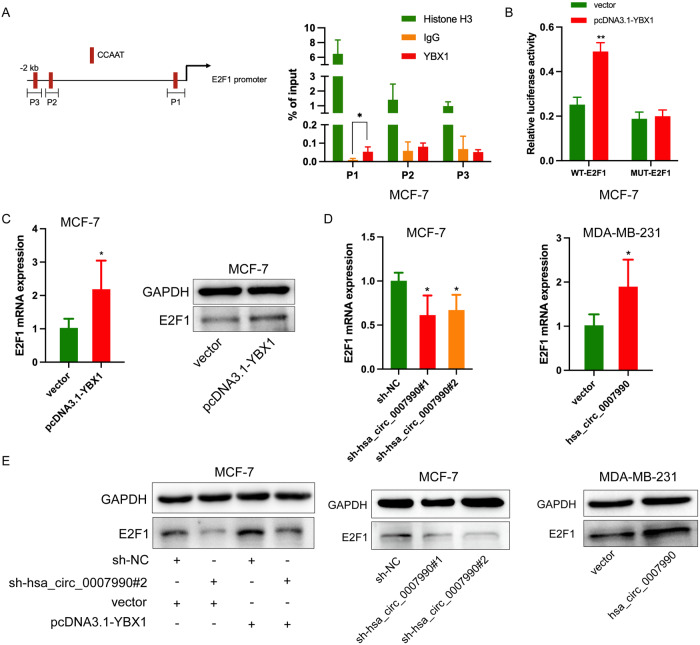
Hsa_circ_0007990 regulates E2F1 expression via a YBX1-dependent pathway. **A** ChIP-qPCR assays revealed that YBX1 protein mainly bound to the Y-box sequence at the −135 to −139 promoter region of E2F1. **B** Luciferase reporter assays indicated that YBX1 interacted with the E2F1 promoter region directly. **C** The mRNA and protein levels of E2F1 were measured by qPCR and western blot after YBX1 overexpression in MCF-7 cells. **D** The mRNA and protein levels of E2F1 were measured by qPCR and western blot after silence and overexpression of hsa_circ_0007990. **E** Rescue experiments showed that elevated YBX1 expression could reverse the decreased E2F1 levels caused by hsa_circ_0007990 knockdown. *P < 0.05, **P < 0.01.

The YBX1 protein can perform multiple physiological functions both in the cytoplasm and inside the nucleus. Nuclear YBX1 functions mainly as a transcription factor [20, 21]. E2F1, a transcript well known for its ability to control cell proliferation [22], has been demonstrated that YBX1 could transcriptionally activate its promoter in MCF-7 cells [23]. Previous studies have reported that YBX1 usually binds to a specific sequence called the Y-box (CCAAT) in target gene promoters [24]. To identify the exact binding sites for YBX1 in the E2F1 promoter region, we searched the −1 to −2 kb upstream of the transcription start site (TSS) in the proximal promoter region of human E2F1 and found three Y-box sequences (Fig. 8A, left). Specific primers for these three target fragments were designed for ChIP-qPCR (Supplementary Table S1). The results revealed that YBX1 protein mainly bound to the Y-box sequence at the −135 to −139 promoter region of E2F1 (Fig. 8A, right). In addition, luciferase reporter assays performed on the −135 to −139 promoter region of E2F1 indicated that YBX1 interacted with the E2F1 promoter region directly (Fig. 8B). Additionally, the overexpression of YBX1 in MCF-7 cells increased E2F1 mRNA and protein levels (Fig. 8C). Bioinformatic analysis revealed that the expression of YBX1 was positively correlated with that of E2F1 (Supplementary Fig. S7). Furthermore, we investigated the effect of hsa_circ_0007990 on E2F1 expression. The results showed that the mRNA and protein levels of E2F1 changed with hsa_circ_0007990 knockdown or overexpression (Fig. 8D). Rescue experiments exhibited that E2F1 overexpression could partly reverse the decreased ability of cell proliferation caused by hsa_circ_0007990 knockdown (Supplementary Fig. S8). Moreover, elevated YBX1 expression reversed the decreased E2F1 levels caused by hsa_circ_0007990 knockdown (Fig. 8E). The IHC and ISH results for 40 BC tissues from the same tumour tissue microarray revealed that the level of hsa_circ_0007990 was correlated with the YBX1 protein (*P* = 0.027) and E2F1 mRNA (*P* = 0.038) levels, respectively (Supplementary Fig. S9). These results suggested that hsa_circ_0007990 might regulate E2F1 expression via a YBX1-dependent pathway.

## Discussion

**Fig. 9:**
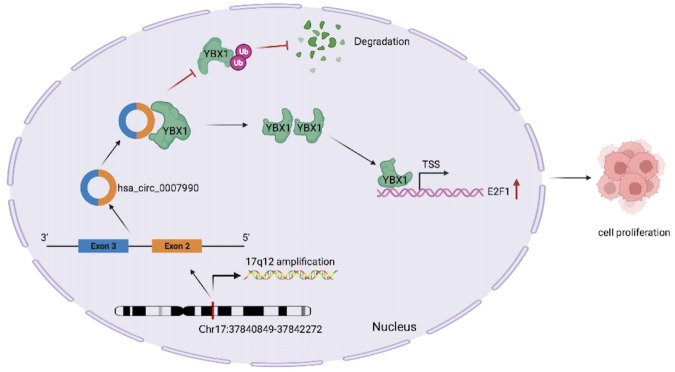
Schematic illustration of the potential function of hsa_circ_0007990 in BC progression. Hsa_circ_0007990 interacted with YBX1 and inhibited its ubiquitination and degradation, subsequently promoted E2F1 transcription.

With the rapid development of RNA sequencing (RNA-seq) of non-polyadenylated transcriptomes, circRNAs have been extensively identified and attracted great interest. Due to the majority of circRNAs originate from exons circularization of pre-mRNA in host gene, genomic aberrations may result in abnormal circRNA expression. In the present study, we focused on the novel circRNA hsa_circ_0007990 in BC through the integrated analysis of circRNA high-throughput sequencing and somatic CNV data. Our results revealed that hsa_circ_0007990 is significantly upregulated in BC partly due to the amplification of the host gene PGAP3. The knockdown of hsa_circ_0007990 suppressed BC cell growth. Mechanistically, hsa_circ_0007990 interacted with YBX1 and inhibited its ubiquitination and degradation, subsequently promoted E2F1 transcription (Fig. 9). Our research indicated that hsa_circ_0007990 is a crucial factor controlling the progression of BC.

In our work, hsa_circ_0007990 was shown to be originate from exons 2 and 3 of the host gene PGAP3, which encodes a glycosylphosphatidylinositol (GPI)-specific phospholipase. Specific amplification of the locus harbouring PGAP3 has been detected in breast carcinoma samples [25], and the identification of the CNV of PGAP3 is important for the accurate diagnosis of BC subtypes [26]. Additionally, our qPCR results showed that the PGAP3 copy number was gained in MCF-7 and T47D cells. The same host gene could produce many different circRNAs due to the alternative circularization [3]. Tian et al. reported that hsa_circ_0106800, which is derived from PGAP3, suppressed cervical cancer tumorigenesis by regulating the miR-769-5p/p53 axis [27]. Interestingly, He et al. unveiled that hsa_circ_0106800 generated from PGAP3 promotes the proliferation and invasion of triple-negative breast cancer by regulating the miR-330-3p/Myc axis [28]. However, our data suggested that hsa_circ_0007990 produced from PGAP3 functions as a proto-oncogene involved in promoting the proliferation of BC cells. Notably, hsa_circ_0007990 has been identified as a blood biomarker for unruptured intracranial aneurysm with aneurysm wall enhancement [29].

Recent studies have reported that circRNAs participate in biological processes via multiple regulatory mechanisms. Among the various known mechanisms, the function of circRNAs as miRNA sponges has been widely investigated [30]. However, the miRNAs-regulatory function is usually performed by circRNAs distributed in the cytoplasm. Our results showed that hsa_circ_0007990 was predominantly located in the nucleus and thus it seems unlikely that it functions mainly as a miRNA sponges. Recently, increasing attention has focused on the translation function of circRNAs [17]. Bioinformatic analysis revealed that hsa_circ_0007990 has no IRES, suggesting that the possibility of this circRNA encoding a protein is relatively low. Additionally, circRNAs can exert intrinsic functions by interacting with proteins and transcription factors [31]. In our study, MS2 RNA pull-down and mass spectrometry assays were conducted to identify that YBX1 protein could bind to hsa_circ_0007990 in BC. As a multifunctional RNA-binding protein, YBX1 has been demonstrated to mediate BC progression by interacting with RNA transcripts, including endogenous tRNA-derived fragments [32], mRNAs [33, 34] and long non-coding RNAs [35–37]. Additionally, YBX1 can affect the tumour development by binding to circRNAs. Chen et al. indicated that circACTN4 recruits YBX1 to initiate FZD7 transcription, which contributes to intrahepatic cholangiocarcinoma progression [38]. Similarly, circIPO7 binds to YBX1 and facilitates its nuclear translocation, which promotes nasopharyngeal carcinoma metastasis and cisplatin chemoresistance [39]. Survival analysis in this study revealed that BC patients with higher YBX1 expression had a poor prognosis. Elevated YBX1 expression could partially rescue the inhibition of cell proliferation caused by hsa_circ_0007990 knockdown. Thus, our work suggested that hsa_circ_0007990 invokes BC progression through interacting with YBX1. Previous studies have shown that degradation of YBX1 protein can be modulated by circRNAs. For example, circRNA-SORE binds to YBX1 protein and subsequently blocks PRP19-mediated YBX1 degradation to promote sorafenib resistance in hepatocellular carcinoma [40]. CircNEIL3 inhibits tumour metastasis by recruiting the E3 ubiquitin ligase Nedd4L to degrade YBX1 [41]. In this study, we demonstrated that hsa_circ_0007990 might stabilize YBX1 protein by preventing its ubiquitin/proteasome-dependent protein degradation.

E2F1 is a driver oncogene involved in the occurrence and development of malignant tumours. Numerous studies have demonstrated that the overexpression of E2F1 contributes to the abnormal growth of cells [42, 43]. Lasham et al. reported that YBX1 may control tumour cell growth through a process associated with the activity of E2F transcription [23]. Razpotnik et al. identified an upregulated circRNA, hsa_circ_0062682, that could interact with YBX1 in hepatocellular carcinoma. Knockdown of hsa_circ_0062682 revealed the transcription factors E2F1, Sp1, HIF-1α, and NFκB1 as potential downstream targets [44]. However, this study did not explore the relationship between YBX1 and E2F1. In our research, ChIP-qPCR and luciferase reporter assays revealed that YBX1 protein bound mainly to the Y-box sequence at the −135 to −139 bp promoter region of E2F1. Moreover, overexpression of YBX1 in MCF-7 cells increased E2F1 mRNA and protein levels. Furthermore, the mRNA and protein levels of E2F1 changed with hsa_circ_0007990 knockdown or overexpression. Elevated YBX1 expression reversed the decreased E2F1 levels caused by hsa_circ_0007990 knockdown. Taken together, these findings suggested that hsa_circ_0007990 might regulate E2F1 expression via a YBX1-dependent pathway.

In conclusion, our study identified and characterized a novel circRNA hsa_circ_0007990, which was upregulated in BC cells partially due to the amplification of the host gene PGAP3. The results also showed that hsa_circ_0007990 was overexpressed in BC tissues and associated with malignant progression in BC patients although the difference was not significant due to the small sample size. Functionally, hsa_circ_0007990 promoted the proliferation and tumorigenesis of BC in vitro and vivo. Mechanistically, hsa_circ_0007990 interacted with YBX1 and inhibited its ubiquitination and degradation, subsequently promoted E2F1 transcription. Our investigation illustrated the possible role of hsa_circ_0007990 in BC and provided a promising therapeutic target for the treatment of BC.

### Supplementary information

Supplementary Results

Supplement Table

Original western blots

aj-checklist

## Data Availability

The data sets used and/or analyzed during the current study are available from the corresponding author on reasonable request.
