# Supplementary material for: Hsa_circ_0007990 promotes breast cancer growth via inhibiting YBX1 protein degradation to activate E2F1 transcription
Source: Cell Death Dis. 2024 Feb 20;15(2):153. doi: 10.1038/s41419-024-06527-7 (PMC10879541; doi:10.1038/s41419-024-06527-7)
Supplement: Supplementary file 1 — Supplementary Results [file 41419_2024_6527_MOESM1_ESM.docx]

Figure S1


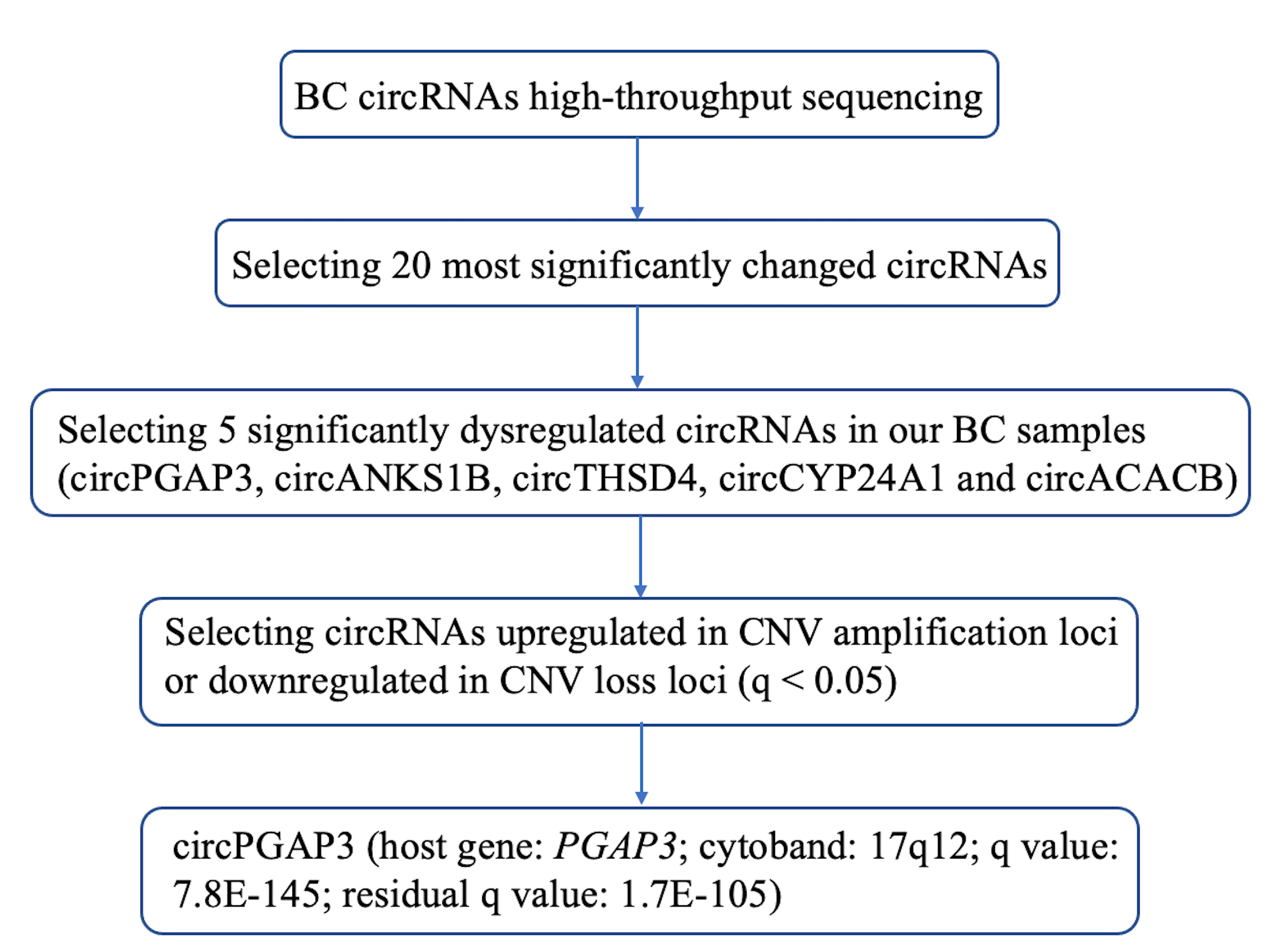


Fig. S1 Screening flowchart of circPGAP3.

Figure S2


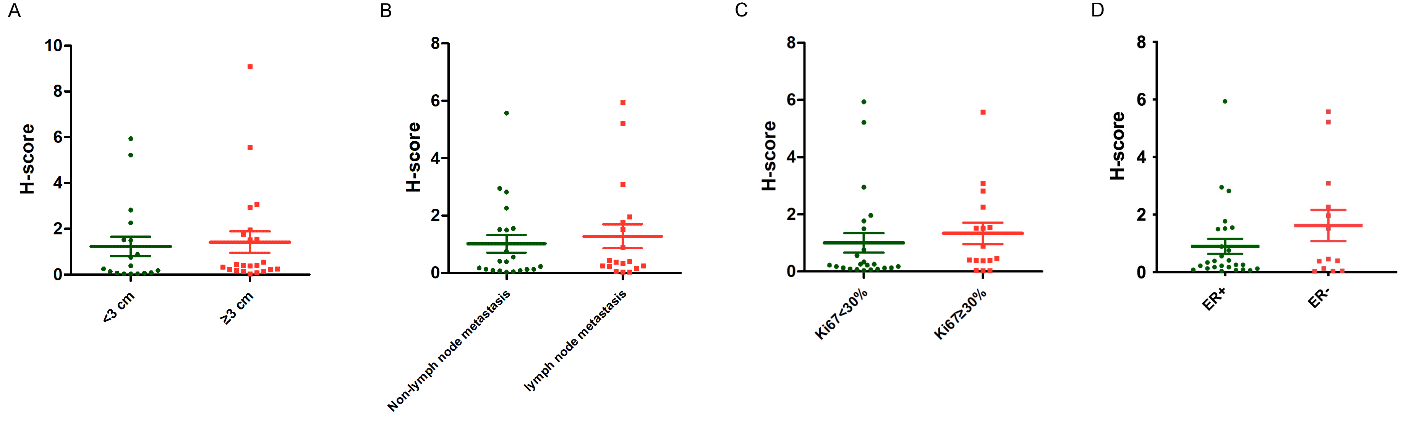


Fig. S2 Elevated expression of hsa_circ_0007990 was associated with BC progression. The expression of hsa_circ_0007990 in BC patients (A) with tumor size < 3 cm and ≥ 3 cm, (B) with or without lymph node metastasis, (C) with Ki67 < 30% and ≥ 30%, (D) with ER+ and ER-.

Figure S3


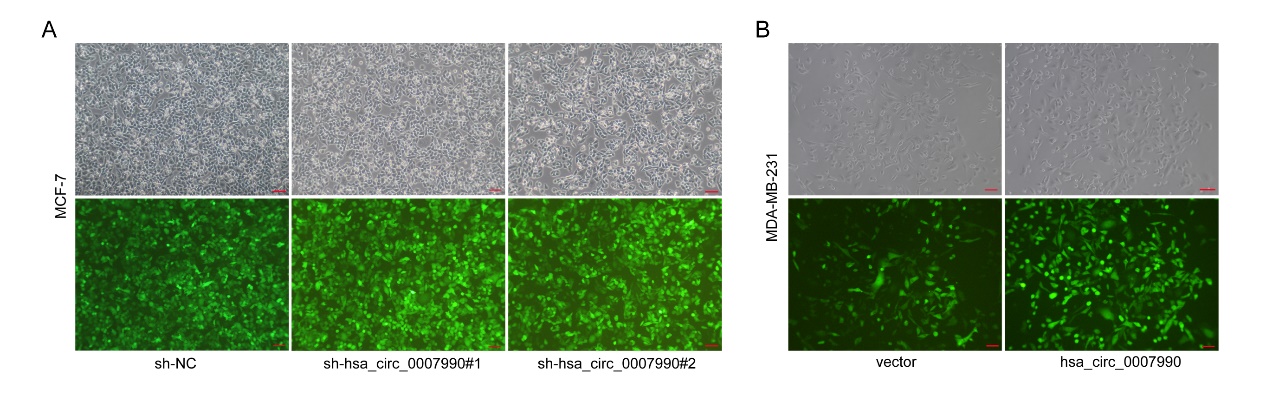


Fig. S3 Representative fluorescent images showed the efficiency of BC cells transfected with (A) sh-hsa_circ_0007990, (B) overexpression vector, and corresponding controls through lentivirus. Scale bar = 50 μm.

Figure S4


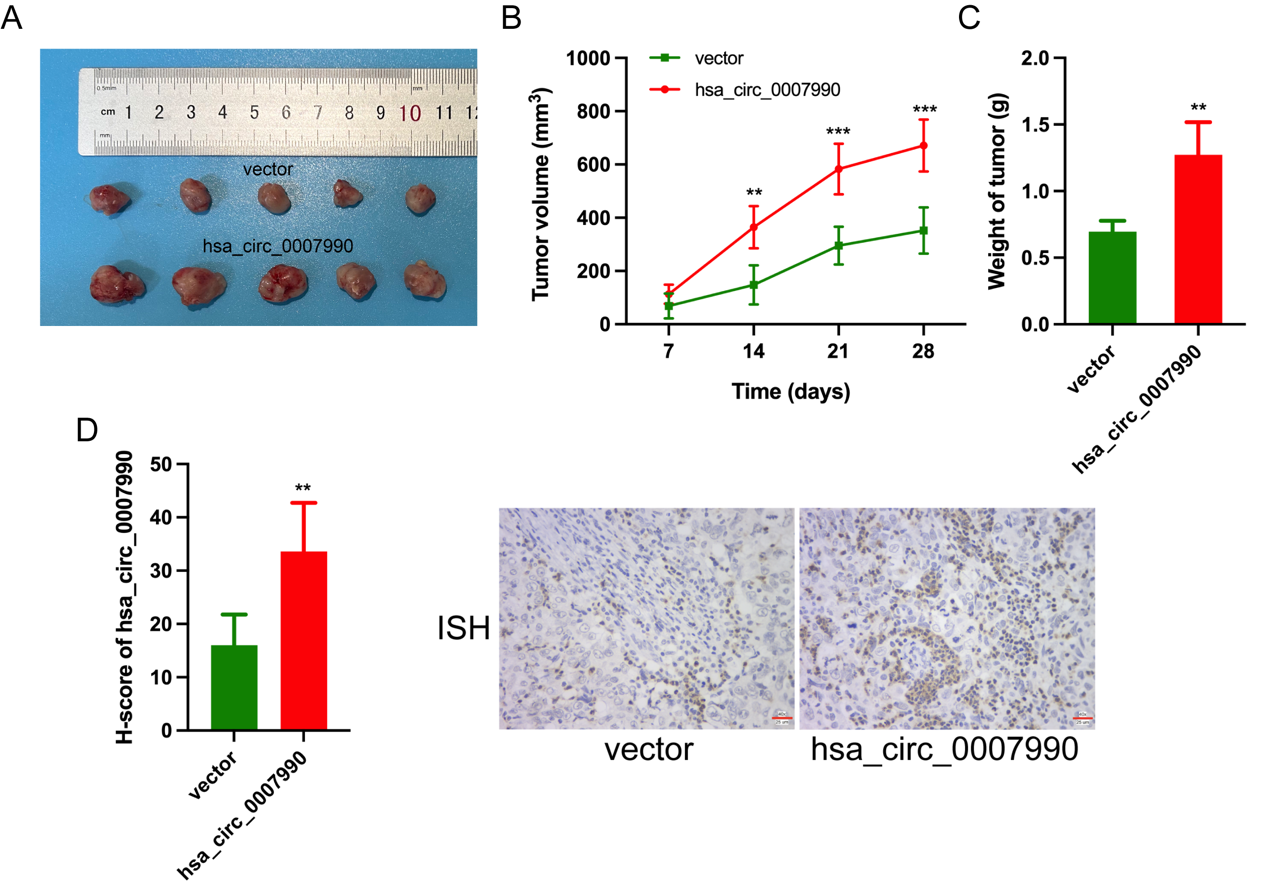


Fig. S4 Hsa_circ_0007990 promotes the growth of BC in vivo. (A) Tumor xenograft model in nude mice (MDA-MB-231 cells). (B) A faster growth rate and (C) higher tumor weight was observed in the xenograft tumors treated with hsa_circ_0007990 overexpression compared with the control group. (D) Representative images for ISH analysis detecting hsa_circ_0007990 expression of xenograft tumors in different groups. Sample size n = 5 for each group. Scale bar = 25 μm. * *P* < 0.05, ** *P* < 0.01, *** *P* < 0.001.

Figure S5


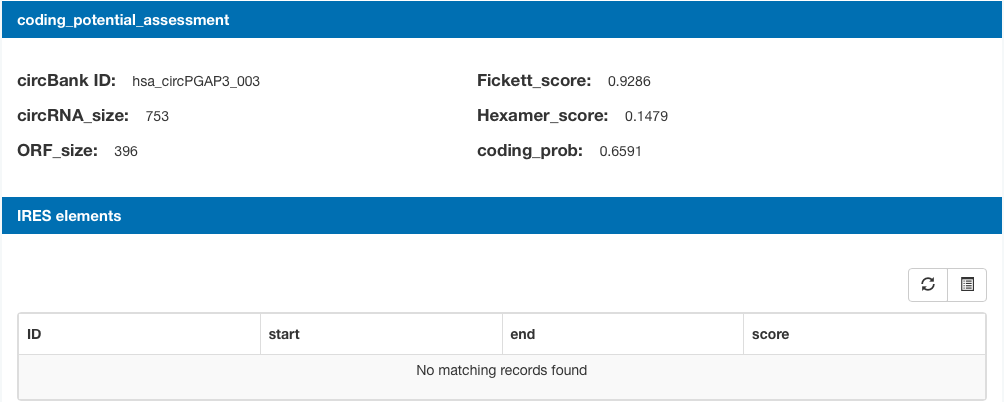


Fig. S5 Bioinformatic analysis by circBank (<http://www.circbank.cn>) showed that hsa_circ_0007990 has no IRES.

Figure S6


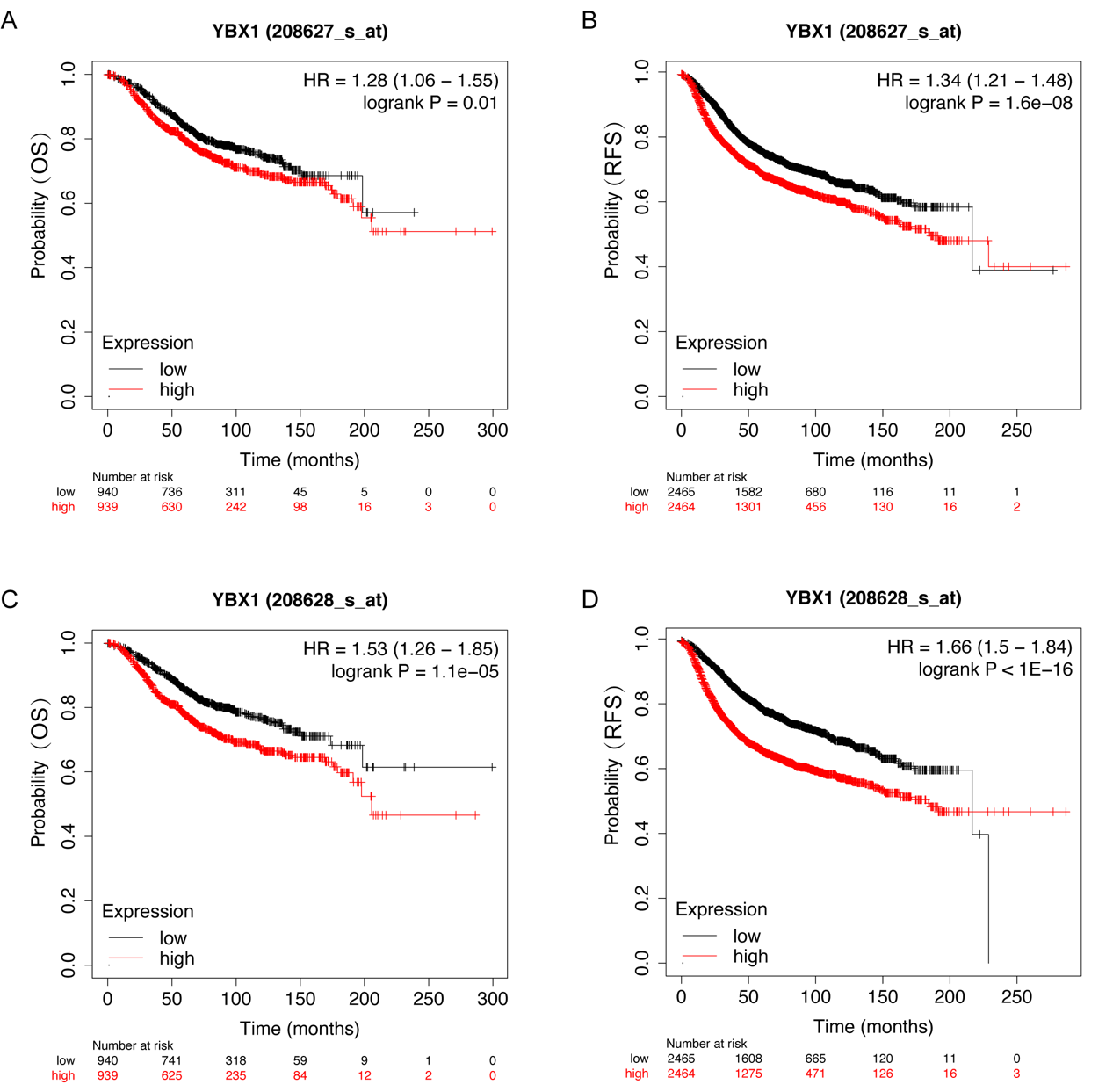


Fig. S6 Kaplan-Meier plotter analysis showed that YBX1 (Affymetrix ID: 208627_s_at and 208628_s_at) expression was associated with poor (A, C) overall survival and (B, D) recurrence free survival in BC patients.

Figure S7

Fig. S7 Bioinformatic analysis showed that the expression of YBX1 positively correlated with E2F1 (data from GTBAdb).

Figure S8


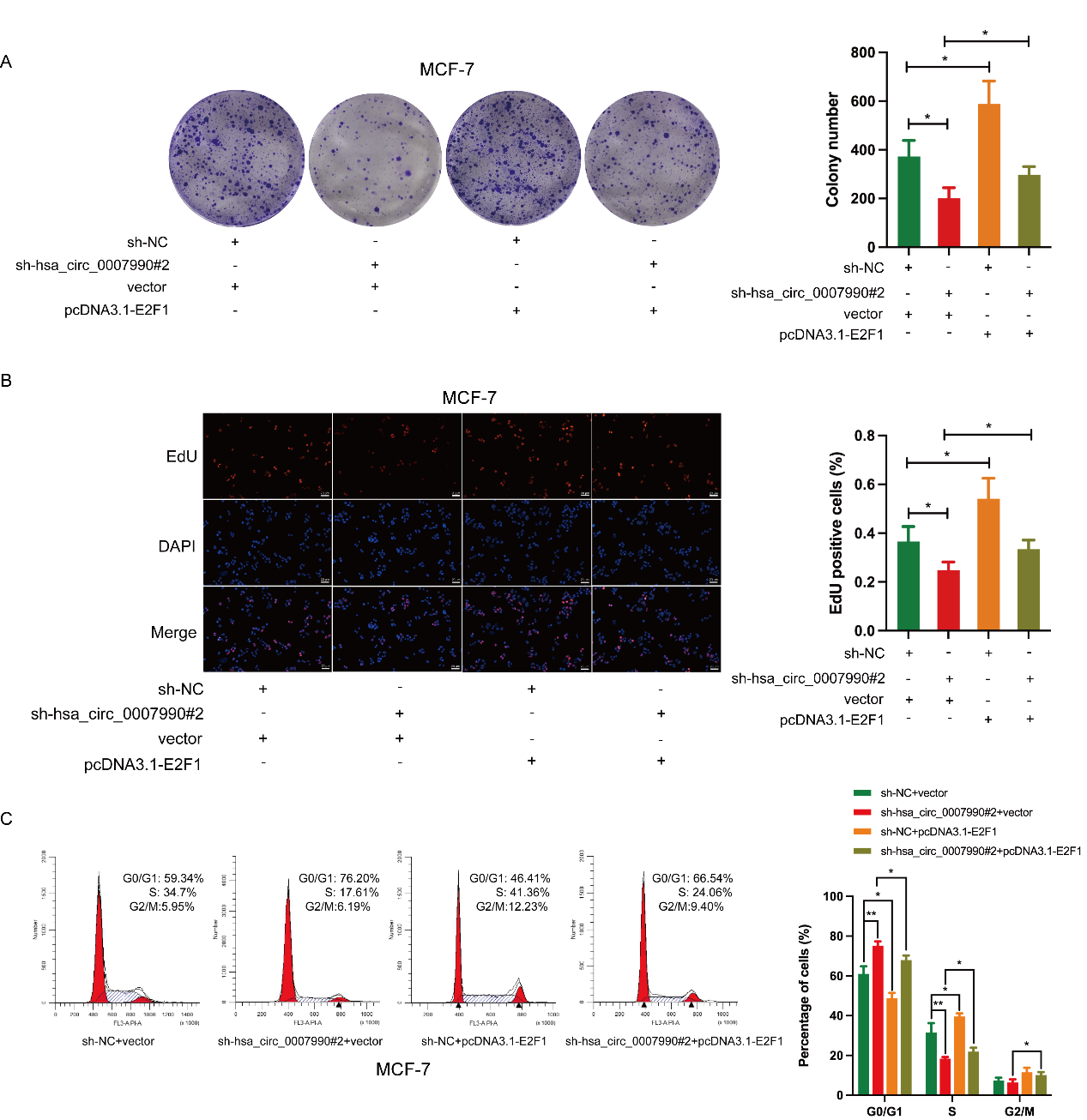


Fig. S8 Rescue experiments performed by (**A**) colony formation, (**B**) EdU, and (**C**) cell cycle assays exhibited that E2F1 overexpression could partly reverse the decreased ability of cell proliferation caused by hsa_circ_0007990 knockdown. Scale bar = 20 μm. * *P* < 0.05, ** *P* < 0.01.

Figure S9


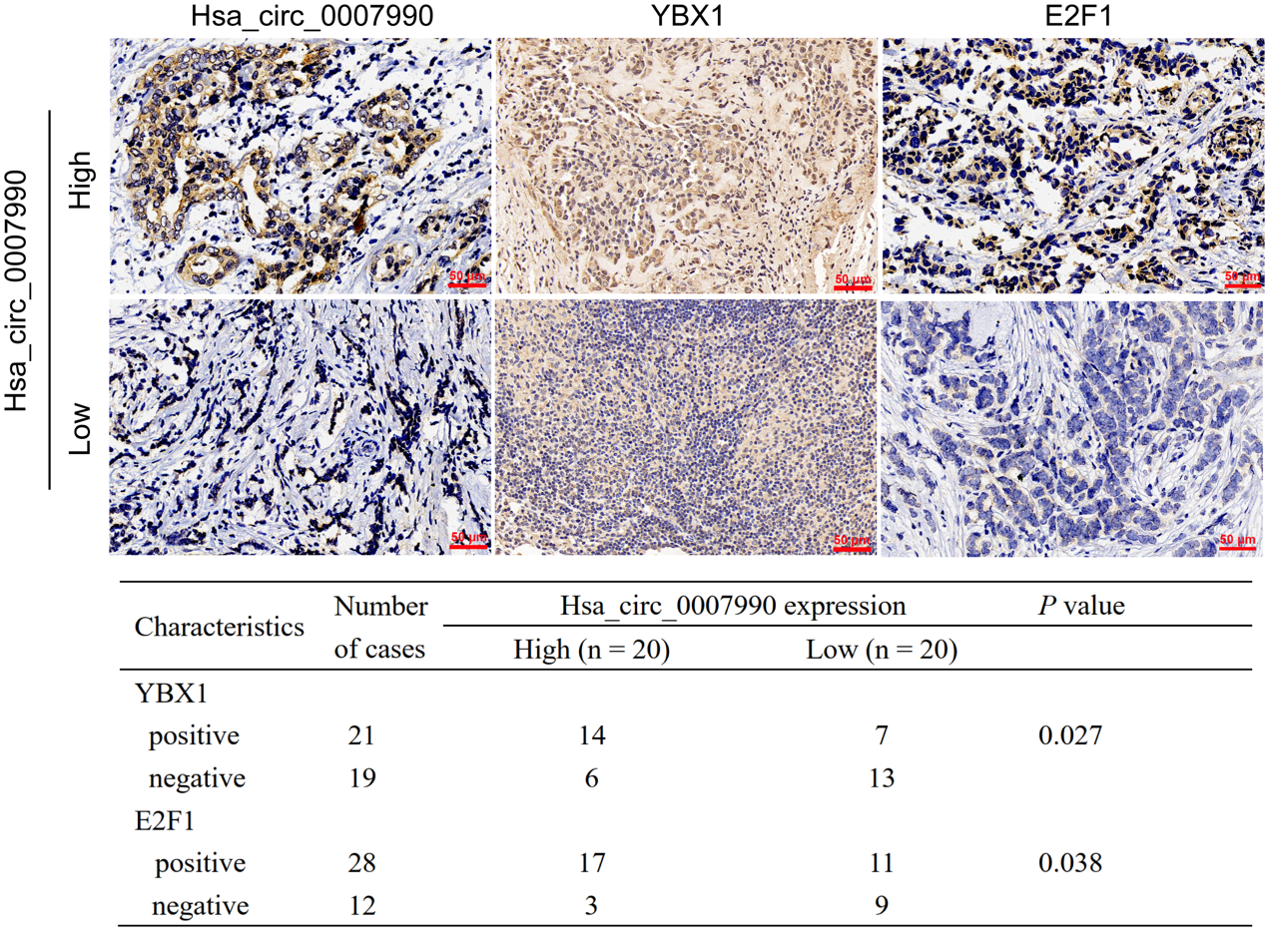


Fig. S9 Analysis of the correlation between YBX1 protein, E2F1 mRNA and hsa_circ_0007990 in 40 BC tissues. The level of hsa_circ_0007990 were correlated with YBX1 protein (*P* = 0.027) and E2F1 mRNA (*P* = 0.038), respectively. Scale bar = 50 μm.
