# Supplementary material for: Hsa_circ_0007990 promotes breast cancer growth via inhibiting YBX1 protein degradation to activate E2F1 transcription
Source: Cell Death Dis. 2024 Feb 20;15(2):153. doi: 10.1038/s41419-024-06527-7 (PMC10879541; doi:10.1038/s41419-024-06527-7)
Supplement: Supplementary file 2 — Supplement Table [file 41419_2024_6527_MOESM2_ESM.docx]

**Table S1. The detailed primers, probes and sh-RNA sequences used in this study.**

| **qRT-PCR primers** | | |
| --- | --- | --- |
| **Gene** | **Forward Primer** | **Reverse Primer** |
| GAPDH | 5′-GCCGTCTAGAAAAACCTGCC-3′ | 5′-CCACCTGGTGCTCAGTGTAG-3′ |
| hsa_circ_0007990 | 5′-TCCTCCCCCATGTACCACAC-3′ | 5′-GCCATGGAACTGAGGCACTT-3′ |
| PGAP3 | 5′-ATCTTGAGGGACCAAGCTGC-3′ | 5′-GGCCCTGAAAGGGAGTATGG-3′ |
| circGAPDH | 5′-GTGCTCAACCAGTTAGCTCTC-3′ | 5′-CCAAATCCGTTGACTCCGAC-3′ |
| U6 | 5′-CTCGCTTCGGCAGCACA-3′ | 5′-AACGCTTCACGAATTTGCGT-3′ |
| YBX1 | 5'-CAAGGTAGACCAGTGAGGCAGAATATG-3' | 5'-TCTAGGCTGTCTTTGGCGAGGAG-3' |
| E2F1 | 5'-CTGTGCCCTGAGGAGACCGTAG-3' | 5'-GATGATGGTGGTGGTGACACTATGG-3' |
| **primers for copy number detection** | | |
| **Gene** | **Forward Primer** | **Reverse Primer** |
| PGAP3-DNA-1 | 5′-GTACCGCGACTGCGTACTG-3′ | 5′-CTCATGTAGATTGGCTGGCGG-3′ |
| PGAP3-DNA-2 | 5′-GTTGCCTGCCCTTCAAACCT-3′ | 5′-GCCATTGAGAAACGAGGCCA-3′ |
| PGAP3-DNA-3 | 5′-CATTGCCCTAGCCAGGTTCC-3′ | 5′-AGGCTCTCAACCAGACAGCA-3′ |
| **primers for ChIP-qPCR** | | |
| **Gene** | **Forward Primer** | **Reverse Primer** |
| E2F1-P1 | 5′-ACTCATTGGAGGGGAAAACAACT-3′ | 5′-TTCAAATGGGCACACAGACCT-3′ |
| E2F1-P2 | 5′-CGCCCCATCCCGCCCCTCG-3′ | 5′-ATCCTTTTTGCCGCGAAAGAGCC-3′ |
| E2F1-P3 | 5′-CCGGCGCGTTAAAGCCAATAGGA-3′ | 5′-TTTTGCCGCGAAAGAGCCACGAG-3′ |
| **sh-RNA sequences** | | |
| sh-hsa_circ_0007990#1 | CTTCGCCTGGGCTGGACCTGT | |
| sh-hsa_circ_0007990#2 | GCCTTCGCCTGGGCTGGACCT | |

**Table S2. Mass spectrometry results of MS2 RNA pull-down assay.**

| prot_hit_num | prot_acc | prot_desc | prot_score | prot_mass | prot_matches | prot_matches_sig | prot_sequences | prot_sequences_sig | prot_cover | prot_pi | emPAI |
| --- | --- | --- | --- | --- | --- | --- | --- | --- | --- | --- | --- |
| 14 | sp\|P60709\|ACTB_HUMAN | Actin, cytoplasmic 1 OS=Homo sapiens OX=9606 GN=ACTB PE=1 SV=1 | 195 | 42052 | 8 | 8 | 7 | 7 | 21.3 | 5.29 | 0.83 |
| 16 | sp\|P10809\|CH60_HUMAN | 60 kDa heat shock protein, mitochondrial OS=Homo sapiens OX=9606 GN=HSPD1 PE=1 SV=2 | 143 | 61187 | 3 | 3 | 1 | 1 | 3.8 | 5.7 | 0.05 |
| 17 | sp\|P67809\|YBOX1_HUMAN | Y-box-binding protein 1 OS=Homo sapiens OX=9606 GN=YBX1 PE=1 SV=3 | 122 | 35903 | 2 | 2 | 2 | 2 | 11.1 | 9.87 | 0.19 |
| 20 | sp\|P25705\|ATPA_HUMAN | ATP synthase subunit alpha, mitochondrial OS=Homo sapiens OX=9606 GN=ATP5F1A PE=1 SV=1 | 90 | 59828 | 1 | 1 | 1 | 1 | 2.7 | 9.16 | 0.05 |
| 22 | sp\|P14618\|KPYM_HUMAN | Pyruvate kinase PKM OS=Homo sapiens OX=9606 GN=PKM PE=1 SV=4 | 79 | 58470 | 2 | 2 | 1 | 1 | 3 | 7.96 | 0.12 |
| 23 | sp\|A0A075B6S2\|KVD29_HUMAN | Immunoglobulin kappa variable 2D-29 OS=Homo sapiens OX=9606 GN=IGKV2D-29 PE=3 SV=1 | 78 | 13249 | 2 | 2 | 2 | 2 | 16.7 | 6.54 | 0.58 |
| 24 | sp\|P06733\|ENOA_HUMAN | Alpha-enolase OS=Homo sapiens OX=9606 GN=ENO1 PE=1 SV=2 | 75 | 47481 | 2 | 2 | 1 | 1 | 4.6 | 7.01 | 0.07 |
| 26 | sp\|P05141\|ADT2_HUMAN | ADP/ATP translocase 2 OS=Homo sapiens OX=9606 GN=SLC25A5 PE=1 SV=7 | 61 | 33059 | 2 | 2 | 2 | 2 | 8.1 | 9.71 | 0.21 |
| 27 | sp\|Q71DI3\|H32_HUMAN | Histone H3.2 OS=Homo sapiens OX=9606 GN=H3C15 PE=1 SV=3 | 60 | 15436 | 5 | 5 | 3 | 3 | 19.1 | 11.27 | 0.82 |
| 28 | sp\|Q02413\|DSG1_HUMAN | Desmoglein-1 OS=Homo sapiens OX=9606 GN=DSG1 PE=1 SV=2 | 59 | 114702 | 1 | 1 | 1 | 1 | 1 | 4.9 | 0.03 |
| 30 | sp\|P04406\|G3P_HUMAN | Glyceraldehyde-3-phosphate dehydrogenase OS=Homo sapiens OX=9606 GN=GAPDH PE=1 SV=3 | 55 | 36201 | 1 | 1 | 1 | 1 | 6.3 | 8.57 | 0.09 |
| 31 | sp\|Q9BQE3\|TBA1C_HUMAN | Tubulin alpha-1C chain OS=Homo sapiens OX=9606 GN=TUBA1C PE=1 SV=1 | 51 | 50548 | 1 | 1 | 1 | 1 | 3.3 | 4.96 | 0.07 |
| 33 | sp\|P29401\|TKT_HUMAN | Transketolase OS=Homo sapiens OX=9606 GN=TKT PE=1 SV=3 | 50 | 68519 | 1 | 1 | 1 | 1 | 4.2 | 7.58 | 0.05 |
| 36 | sp\|P18621\|RL17_HUMAN | 60S ribosomal protein L17 OS=Homo sapiens OX=9606 GN=RPL17 PE=1 SV=3 | 39 | 21611 | 1 | 1 | 1 | 1 | 8.7 | 10.18 | 0.16 |
| 39 | sp\|Q93070\|NAR4_HUMAN | Ecto-ADP-ribosyltransferase 4 OS=Homo sapiens OX=9606 GN=ART4 PE=2 SV=2 | 35 | 36197 | 2 | 2 | 1 | 1 | 2.2 | 9.31 | 0.19 |
| 41 | sp\|P42702\|LIFR_HUMAN | Leukemia inhibitory factor receptor OS=Homo sapiens OX=9606 GN=LIFR PE=1 SV=1 | 31 | 125090 | 1 | 1 | 1 | 1 | 0.6 | 5.5 | 0.03 |
| 42 | sp\|P61769\|B2MG_HUMAN | Beta-2-microglobulin OS=Homo sapiens OX=9606 GN=B2M PE=1 SV=1 | 31 | 13820 | 1 | 1 | 1 | 1 | 8.4 | 6.06 | 0.25 |
| 43 | sp\|Q9Y6A4\|CFA20_HUMAN | Cilia- and flagella-associated protein 20 OS=Homo sapiens OX=9606 GN=CFAP20 PE=1 SV=1 | 31 | 22931 | 1 | 1 | 1 | 1 | 6.2 | 9.78 | 0.15 |
| 44 | sp\|Q08554\|DSC1_HUMAN | Desmocollin-1 OS=Homo sapiens OX=9606 GN=DSC1 PE=1 SV=2 | 31 | 101406 | 1 | 1 | 1 | 1 | 1.5 | 5.25 | 0.03 |
| 48 | sp\|Q5SNV9\|CA167_HUMAN | Uncharacterized protein C1orf167 OS=Homo sapiens OX=9606 GN=C1orf167 PE=2 SV=2 | 28 | 164602 | 1 | 1 | 1 | 1 | 0.5 | 10.71 | 0.02 |
| 50 | sp\|O75792\|RNH2A_HUMAN | Ribonuclease H2 subunit A OS=Homo sapiens OX=9606 GN=RNASEH2A PE=1 SV=2 | 27 | 33716 | 1 | 1 | 1 | 1 | 3.3 | 5.14 | 0.1 |
| 51 | sp\|P0C0S5\|H2AZ_HUMAN | Histone H2A.Z OS=Homo sapiens OX=9606 GN=H2AZ1 PE=1 SV=2 | 27 | 13545 | 1 | 1 | 1 | 1 | 7 | 10.58 | 0.25 |
| 52 | sp\|Q9NSN8\|SNTG1_HUMAN | Gamma-1-syntrophin OS=Homo sapiens OX=9606 GN=SNTG1 PE=1 SV=1 | 27 | 59130 | 1 | 1 | 1 | 1 | 2.1 | 6.24 | 0.06 |
| 53 | sp\|Q9BY43\|CHM4A_HUMAN | Charged multivesicular body protein 4a OS=Homo sapiens OX=9606 GN=CHMP4A PE=1 SV=3 | 26 | 25083 | 1 | 1 | 1 | 1 | 3.2 | 4.65 | 0.13 |
| 54 | sp\|Q6NXT1\|ANR54_HUMAN | Ankyrin repeat domain-containing protein 54 OS=Homo sapiens OX=9606 GN=ANKRD54 PE=1 SV=2 | 26 | 32885 | 1 | 1 | 1 | 1 | 2.3 | 5.84 | 0.1 |
| 55 | sp\|P23759\|PAX7_HUMAN | Paired box protein Pax-7 OS=Homo sapiens OX=9606 GN=PAX7 PE=1 SV=4 | 26 | 55427 | 1 | 1 | 1 | 1 | 1.8 | 9.1 | 0.06 |
| 56 | sp\|O43915\|VEGFD_HUMAN | Vascular endothelial growth factor D OS=Homo sapiens OX=9606 GN=VEGFD PE=1 SV=1 | 26 | 42128 | 1 | 1 | 1 | 1 | 4 | 8.16 | 0.08 |
| 57 | sp\|Q5CZC0\|FSIP2_HUMAN | Fibrous sheath-interacting protein 2 OS=Homo sapiens OX=9606 GN=FSIP2 PE=2 SV=4 | 25 | 785878 | 1 | 1 | 1 | 1 | 0.1 | 6.27 | 0 |
| 58 | sp\|A4UGR9\|XIRP2_HUMAN | Xin actin-binding repeat-containing protein 2 OS=Homo sapiens OX=9606 GN=XIRP2 PE=1 SV=2 | 25 | 383888 | 1 | 1 | 1 | 1 | 0.6 | 5.99 | 0.01 |
| 60 | sp\|Q5THJ4\|VP13D_HUMAN | Vacuolar protein sorting-associated protein 13D OS=Homo sapiens OX=9606 GN=VPS13D PE=1 SV=2 | 23 | 495369 | 1 | 1 | 1 | 1 | 0.2 | 6.15 | 0.01 |
| 61 | sp\|Q9UPY3\|DICER_HUMAN | Endoribonuclease Dicer OS=Homo sapiens OX=9606 GN=DICER1 PE=1 SV=3 | 21 | 221279 | 1 | 1 | 1 | 1 | 0.8 | 5.47 | 0.01 |
| 63 | sp\|Q92538\|GBF1_HUMAN | Golgi-specific brefeldin A-resistance guanine nucleotide exchange factor 1 OS=Homo sapiens OX=9606 GN=GBF1 PE=1 SV=2 | 17 | 208367 | 1 | 1 | 1 | 1 | 0.3 | 5.48 | 0.02 |
| 64 | sp\|P52735\|VAV2_HUMAN | Guanine nucleotide exchange factor VAV2 OS=Homo sapiens OX=9606 GN=VAV2 PE=1 SV=2 | 17 | 102478 | 1 | 1 | 1 | 1 | 1 | 6.67 | 0.03 |
| 65 | sp\|Q96FG2\|ELMD3_HUMAN | ELMO domain-containing protein 3 OS=Homo sapiens OX=9606 GN=ELMOD3 PE=1 SV=2 | 14 | 43418 | 1 | 1 | 1 | 1 | 3.7 | 8.11 | 0.08 |
