# Supplementary material for: Hsa_circ_0007990 promotes breast cancer growth via inhibiting YBX1 protein degradation to activate E2F1 transcription
Source: Cell Death Dis. 2024 Feb 20;15(2):153. doi: 10.1038/s41419-024-06527-7 (PMC10879541; doi:10.1038/s41419-024-06527-7)
Supplement: Supplementary file 3 — Original western blots [file 41419_2024_6527_MOESM3_ESM.docx]

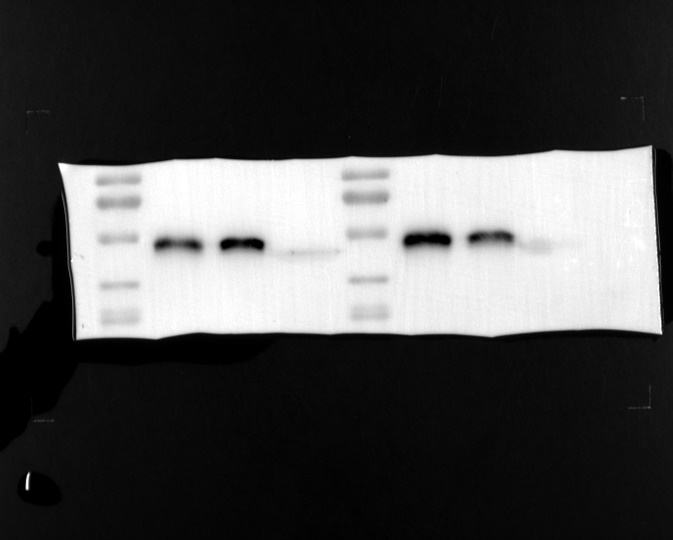


1. This original western blot was used in the paper of Fig. 5B.


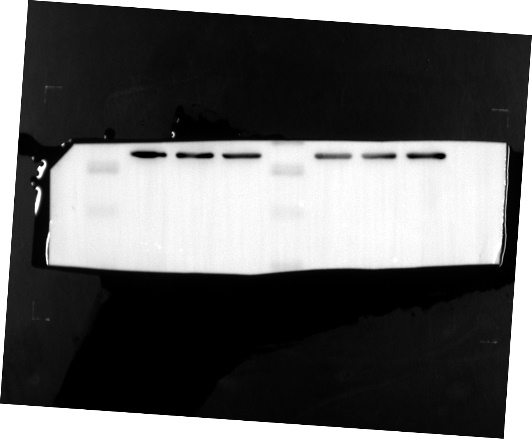

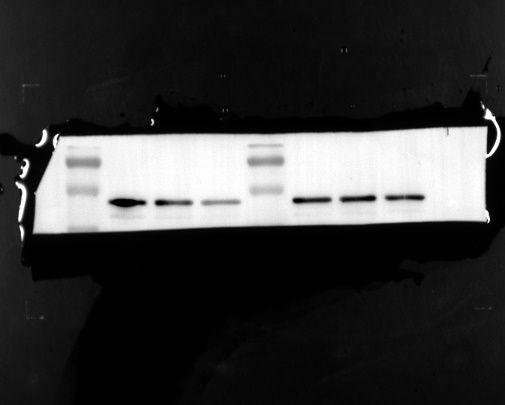


2. The left band is GAPDH, and the right band is YBX1. Three lanes on the left of these two original western blots were used in the paper of Fig. 5E MCF-7 cells.


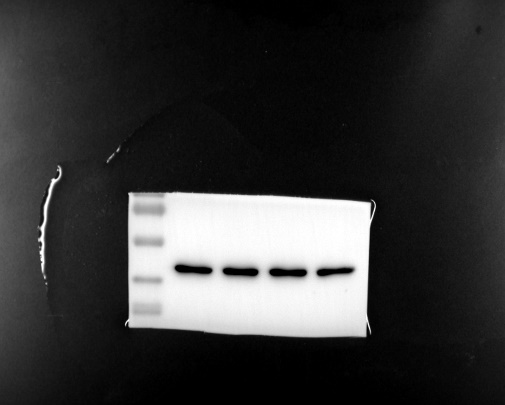

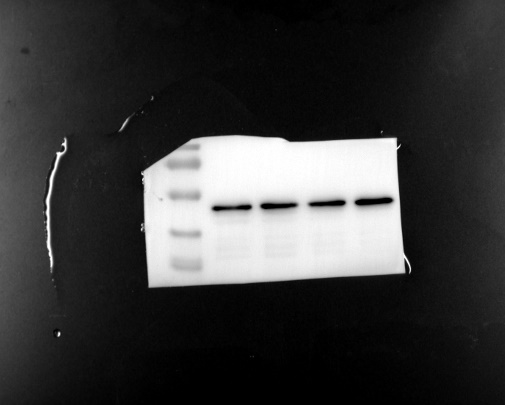


3. The left band is GAPDH, and the right band is YBX1. The lane three and four in these two original western blots were used in the paper of Fig. 5E MDA-MB-231 cells.







4. The left band is GAPDH, and the right band is YBX1. These two original western blots were used in the paper of Fig. 6A sh-hsa_circ_0007990#1.


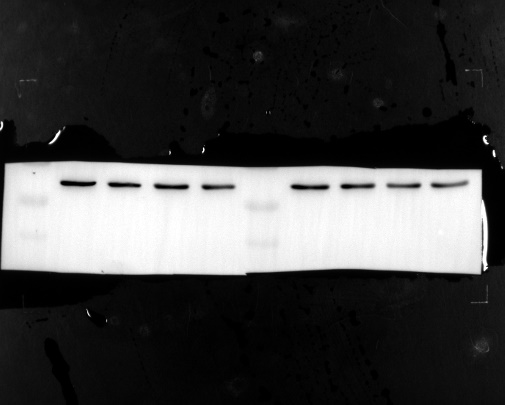

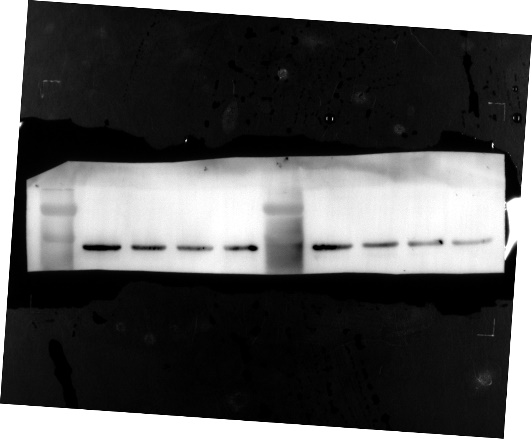


5. The left band is GAPDH, and the right band is YBX1. These two original western blots were used in the paper of Fig. 6A sh-hsa_circ_0007990#2.


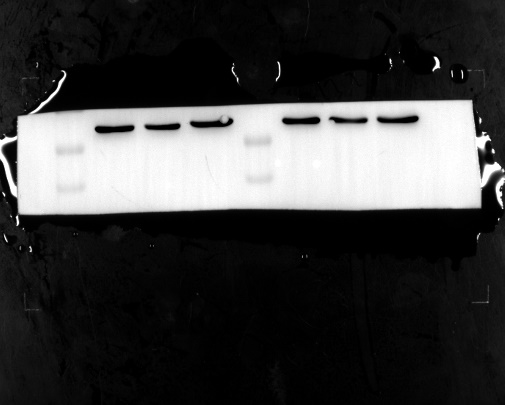

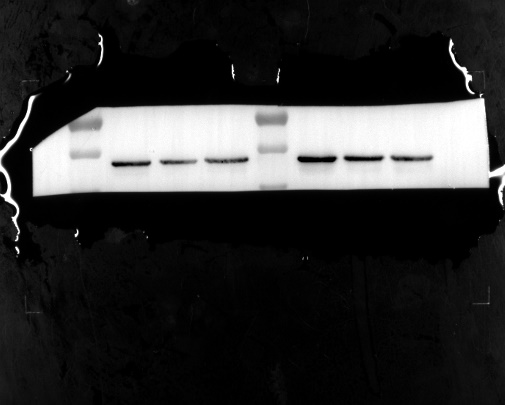


6. The left band is GAPDH, and the right band is YBX1. These two original western blots were used in the paper of Fig. 6B.


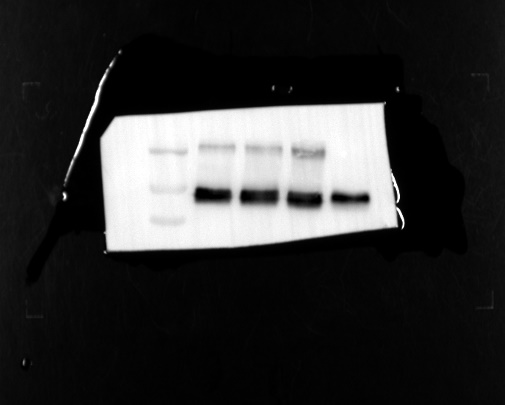

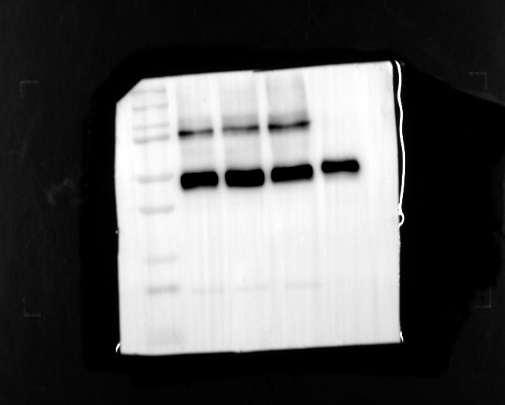


7. The left dark band is Input-YBX1, and the upper band in the right picture is IB-Ubiquitin. The lower dark band in the right picture is IgG heavy chain. These two original western blots were used in the paper of Fig. 6C.


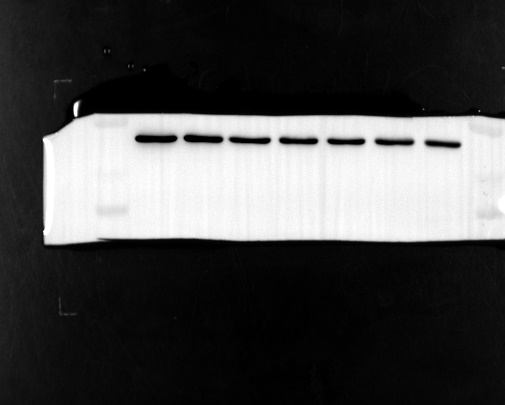

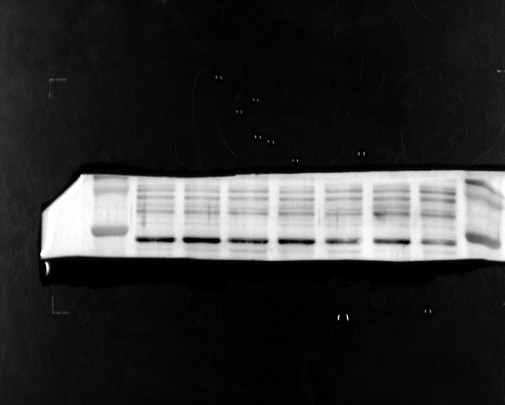


8. The left band is GAPDH, and the right band is YBX1. Two lanes on the left of these two original western blots were used in the paper of Fig. 7A.







9. The left band is GAPDH, and the right band is E2F1. The lane five and six in these two original western blots were used in the paper of Fig. 8C.


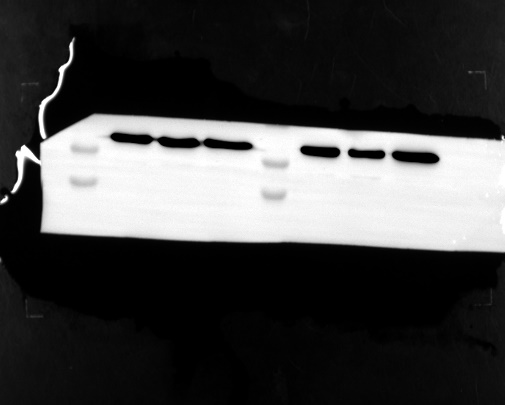

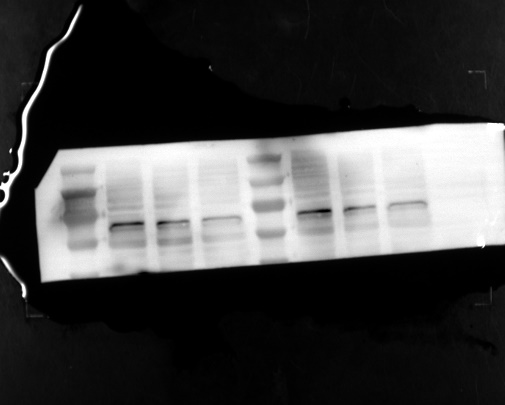


10. The left band is GAPDH, and the right band is E2F1. Three lanes on the right of these two original western blots were used in the paper of Fig. 8D MCF-7 cells.


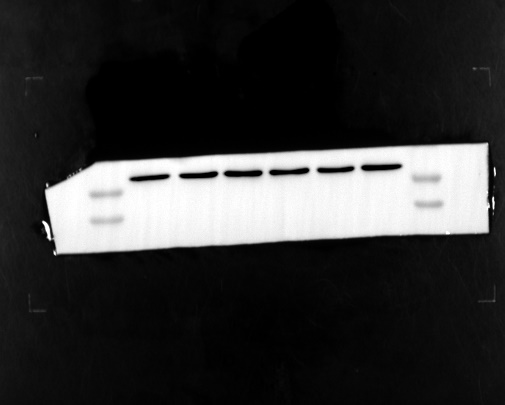




11. The left band is GAPDH, and the right band is E2F1. The lane five and six in these two original western blots were used in the paper of Fig. 8D MDA-MB-231 cells.


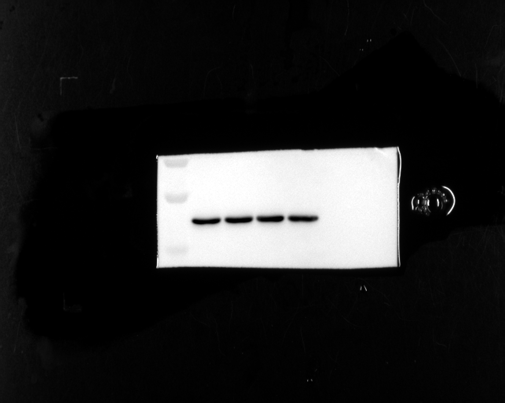

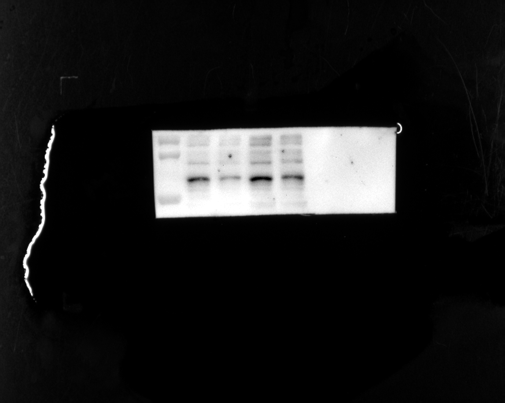


12. These two original western blots were used in the paper of Fig. 8E.
